# Cancer incidence in Stockholm firefighters 1958–2012: an updated cohort study

**DOI:** 10.1007/s00420-017-1276-1

**Published:** 2017-11-21

**Authors:** Cecilia Kullberg, Tomas Andersson, Per Gustavsson, Jenny Selander, Göran Tornling, Annika Gustavsson, Carolina Bigert

**Affiliations:** 1 Unit of Occupational Medicine, Institute of Environmental Medicine, Karolinska Institutet, Stockholm, Sweden; 2 Centre for Occupational and Environmental Medicine, Stockholm County Council, Stockholm, Sweden; 3 Respiratory Medicine Unit, Department of Medicine Solna and CMM, Karolinska Institutet and Karolinska University Hospital, Solna, Sweden

**Keywords:** Occupational exposure, Carcinogens, Particles, Smoke, Firefighters

## Abstract

**Objectives:**

Previous studies on firefighters indicate an increased risk of cancer although findings regarding which cancer sites are in excess have been inconsistent. The aim of this study was to investigate the cancer incidence among Swedish firefighters.

**Methods:**

This updated cohort study included 1080 men who worked at least 1 year as a firefighter in the city of Stockholm, Sweden during 1931–1983. First-time diagnoses of cancer were identified through the Swedish Cancer Registry from 1958 until 2012. Employment as a firefighter was determined from the annual fire station enrolment records. Standardized incidence ratios were calculated using the Stockholm population as reference.

**Results:**

Firefighters in Stockholm had a low overall risk of cancer (SIR = 0.81 95% CI 0.71–0.91). However, firefighters were at an increased risk of stomach cancer (SIR = 1.89 95% CI 1.25–2.75). Firefighters had significantly low risks for prostate cancer (SIR = 0.68 95% CI 0.52–0.87) and malignant melanoma of the skin (SIR = 0.30 95% CI 0.06–0.88). There was a statistically significant trend of increasing overall risk of cancer with increasing employment duration, although there was still no excess of cancer overall in any of the categories of employment duration.

**Conclusion:**

Stockholm firefighters had an increased risk of stomach cancer but a low overall risk of cancer. The trend of increasing overall risk of cancer with increasing employment duration could potentially be related to the carcinogenic exposures at work.

## Introduction

Firefighters are during their work potentially exposed to a wide range of carcinogenic substances. Through the smoke, they may be exposed to carcinogens such as benzene, 1,3-butadiene, formaldehyde and polycyclic aromatic hydrocarbons (PAHs) (IARC 2010). Firefighters could also be exposed to asbestos, crystalline silica and polychlorinated biphenyls (PCBs) depending on the characteristic of the fire site (IARC 2010) as well as to diesel exhaust from the firefighting vehicles (Froines et al. 1987). Additionally, most firefighters work shift which could disrupt the circadian rhythm and potentially increase the risk for some types of cancer (IARC 2010). The occupational exposure may vary between countries, depending on the regulations and use of protective gears, extinguishing methods and other practices used in the occupation. Based on limited evidence in humans and inadequate evidence in experimental animals “occupational exposure as a firefighter” was classified as possibly carcinogenic to humans (Group 2B) by the International Agency for Research on Cancer (IARC) in 2007 (IARC 2010).

Several studies have investigated firefighters’ risk of cancer and some have found an increased risk for various cancer sites. In 2006, LeMasters et al. conducted a review of 32 studies and meta-analysis of 26 studies and found a probably increased risk for multiple myeloma, non-Hodgkin lymphoma, prostate cancer and testicular cancer as well as a possibly increased risk for cancer of the skin, brain, rectum, buccal cavity and pharynx, stomach, colon, leukemia and malignant melanoma (LeMasters et al. 2006). Another comprehensive review and meta-analysis conducted by IARC in 2010 included in addition some studies that were reported after the review by LeMasters et al. and found strongest evidence for increased risk of non-Hodgkin lymphoma, prostate cancer and testicular cancer (IARC 2010). A recent study from the Nordic Occupational Cancer (NOCCA) project including over 16,000 firefighters in the five Nordic countries showed an increased incidence for adenocarcinoma of the lung, prostate cancer, malignant melanoma and non-melanoma skin cancer, as well as an excess risk for all cancer sites combined (Pukkala et al. 2014). Decreased incidence of testicular cancer among firefighters was also observed (Pukkala et al. 2014). Even though there have been many studies on firefighters and cancer conducted, many are limited to information from death certificates which limit the possibility to study cancer sites with high survival rates.

There are a limited number of studies on Swedish firefighters’ risk of cancer. Tornling et al. conducted in 1994 a cohort study on all firefighters who worked in Stockholm, Sweden for at least 1 year during 1931–1983 (Tornling et al. 1994). Cancer incidence was studied from 1958 to 1986. The study showed an increased incidence of stomach cancer and a tendency for increasing incidence of brain- and stomach cancer with increasing number of fires (Tornling et al. 1994). Since this Swedish study was conducted, several international studies have been published indicating an increased cancer risk among firefighters (Daniels et al. 2014; Glass et al. 2016; IARC 2010; LeMasters et al. 2006; Pukkala et al. 2014) which have resulted in increased concern for occupational cancer among the firefighters themselves. Therefore, we decided to update the previous Stockholm cohort study (Tornling et al. 1994) with information on work duration and cancer incidence until 2012, adding 26 years of follow-up. The aim of this study was to investigate the cancer incidence among Swedish firefighters.

## Methods

The study is based on an update and extended follow-up of a previous cohort study described in detail elsewhere (Tornling et al. 1994). The cohort includes men who worked at least 1 year as a firefighter in Stockholm, Sweden during 1931–1983. The cohort was identified through annual enrolment records from 15 fire stations in Stockholm and comprised 1153 men. Of these, 63 had died or emigrated before 1958 when the follow-up began and 10 men had worked less than 1 year, resulting in 1080 men participating in the study.

All men were followed up for cancer incidence from 1 January 1958, when the National Cancer Register was established, to 31 December 2012. This resulted in a follow-up time of 29–54 years, depending on time of employment. All first-time cancers for each specific cancer site were included. The cancer sites were identified using the ICD7-codes (International Classification of Diseases, 7th Revision). By law in Sweden, all malignant tumors are to be reported, and the Cancer Registry has a high coverage of 96% (Barlow et al. 2009). Date of death and migration status were retrieved from population records held by Statistics Sweden.

Information on employment duration was collected through the annual enrolment record listings that were held at each fire station. Information on starting- and ending year of employment for each firefighter was used to calculate employment duration and no data were missing for any of the participants. The employment duration also included years worked before 1931 and years worked until 2012, if they fulfilled the criterion of working at least 1 year between 1931 and 1983. In the analyses, employment duration was classified in 10-year groups, resulting in 1–9, 10–19, 20–29, and ≥ 30 years of employment. The starting year of employment was classified as; 1903–1939, 1940–1959 and 1960–1983. We updated the information on employment duration up until 2012, but unfortunately, updating of data on number of fires fought was not possible to obtain for the extended follow-up.

Ethical approval for this study was obtained by the Stockholm ethical review board (Dnr: 2013/2126-31/3 and 2015/787-32). The study was funded by AFA Insurance in Sweden (Grant no. 130104).

### Statistical analyses

Standardized incidence ratio (SIR) is the observed number of cases in the study population divided by the number of cases expected to occur given the age specific incidence in the reference population. SIRs were calculated for all cancer sites combined and for specific cancer sites one by one. Expected number of cancer cases were obtained from the general male population in Stockholm County by the person year method. The total number of men in the population was about 600,000 in 1958 and just over 1 million in 2012. Standardization was carried out with the reference population of Stockholm County grouped by birth year and calendar year in 5-year intervals. Trends for risk with age, employment duration and starting year of employment were estimated and tested with a log-linear poisson model adjusted for age. Confidence intervals were constructed by the exact method by Garwood (Garwood 1936). We also analysed cancer incidence stratified on age (< 50, 50–64, and ≥ 65 years), employment duration (1–9, 10–19, 20–29, ≥ 30 years) and starting year of employment (1903–1939, 1940–1959, 1960–1983). The analyses were done for the full follow-up (1958–2012) as well as for the former follow-up only (1958–1986) and for the extended follow-up only (1987–2012). In the analyses of the former follow-up only, three new cancer cases were identified (two cases of stomach cancer and one case of brain cancer) that had not been identified in the previous publication. SAS software was used for all statistical analyses.

## Results

The cohort consisted of 1080 Swedish men who were followed up in the cancer registry for up to 54 years, resulting in 265 cancer cases. The firefighters had been working for 1–44 years, with a mean employment duration of 26 years (Table 1). Many firefighters started working early in their lives and the mean age at employment as a firefighter was 25 years.

**Table 1:** Characteristics of Stockholm firefighter cohort

	Min	Max	Mean	Median
Birth year	1881	1960	1925	1925
Age at employment	17	57	25	24
Age at start of follow-up	18	77	38	34
Start of employment	1902	1983	1951	1951
End of employment or follow-up	1933	2012	1977	1977
Employment duration (years)	1	44	26	30

Table 2 presents the observed and expected incidence for all cancer sites with at least two observed cancer cases, sub-divided into three time intervals; for the entire follow-up (1958–2012), for the former follow-up only (1958–1986) and for the extended follow-up only (1987–2012). The total cancer incidence in firefighters was lower than expected (SIR = 0.81 95% CI 0.71–0.91). This was also seen when looking at the extended follow-up only (SIR = 0.67 95% CI 0.56–0.79) but not when looking at the former follow-up only. The risk of stomach cancer was increased in firefighters (SIR = 1.89 95% CI 1.25–2.75) with 27 observed cases among firefighters compared to 14.5 expected cases, something shown also in the former follow-up (SIR = 2.21 95% CI 1.35–3.41). For the extended follow-up only, the power was lower but the tendency towards an increased risk of stomach cancer was present also for this period with 7 observed cases compared to 5.2 expected (SIR = 1.35 95% CI 0.54–2.78). Prostate cancer, on the other hand, showed a significantly decreased risk both in the full follow-up (SIR = 0.68 95% CI 0.52–0.87) and in the extended follow-up only (SIR = 0.48 95% CI 0.33–0.69) but not in the former follow-up. Firefighters also had a low risk for malignant melanoma of the skin in the full follow-up (SIR = 0.30 95% CI 0.06–0.88) as well as in the extended follow-up (SIR = 0.27 95% CI 0.03–0.97). There were no observed cases of testicular cancer among firefighters in the entire follow-up, compared to 1.5 expected cases.

**Table 2:** Observed and expected cancer incidence in 1080 Stockholm firefighters for the full follow-up; 1958–2012, the former follow-up only; 1958–1986 and the extended follow-up only; 1987–2012

Cancer location (ICD 7)	Full follow-up; 1958–2012				Former follow-up only; 1958–1986				Extended follow-up only; 1987–2012			
	Obs^a^	Exp^b^	SIR^c^	95% CI	Obs	Exp	SIR	95% CI	Obs	Exp	SIR	95% CI
Malignant tumor, total (140–209)	265	327.9	0.81	(0.71–0.91)	130	126.0	1.03	(0.86–1.23)	135	201.9	0.67	(0.56–0.79)
Lip (140)	2	1.4	1.45	(0.18–5.26)	1	0.7	1.42	(0.04–7.91)	1	0.7	1.49	(0.38–8.32)
Esophagus (150)	5	5.1	0.99	(0.32–2.30)	1	2.3	0.43	(0.01–2.38)	4	2.7	1.46	(0.40–3.75)
Stomach (151)	27	14.5	1.89	(1.25–2.75)	20	9.1	2.21	(1.35–3.41)	7	5.2	1.35	(0.54–2.78)
Colon (153)	20	23.1	0.86	(0.53–1.34)	8	8.7	0.92	(0.40–1.81)	12	14.4	0.83	(0.43–1.46)
Rectum and anus (154)	18	14.4	1.25	(0.74–1.98)	10	5.8	1.74	(0.83–3.19)	8	8.6	0.93	(0.40–1.82)
Biliary passages of liver (155)	7	8.8	0.79	(0.32–1.63)	4	4.4	0.90	(0.25–2.31)	3	4.4	0.68	(0.14–2.00)
Pancreas (157)	10	9.5	1.06	(0.51–1.94)	6	4.9	1.23	(0.45–2.68)	4	4.6	0.87	(0.24–2.23)
Bronchus and lung primary (1621)	27	34.0	0.79	(0.52–1.15)	17	17.6	0.96	(0.56–1.55)	10	16.4	0.61	(0.29–1.12)
Pleura (1622)	2	0.8	2.41	(0.29–8.71)	1	0.2	5.24	(0.13–29.19)	1	0.64	1.57	(0.04–8.73)
Prostate (177)	60	88.6	0.68	(0.52–0.87)	29	24.3	1.19	(0.80–1.72)	31	64.3	0.48	(0.33–0.69)
Kidney (180)	6	10.6	0.57	(0.21–1.23)	2	5.4	0.37	(0.04–1.33)	4	5.2	0.78	(0.21–1.99)
Urinary organs (181)	16	22.3	0.72	(0.41–1.17)	8	8.4	0.95	(0.41–1.88)	8	13.9	0.58	(0.25–1.14)
Malignant melanoma of skin (190)	3	10.0	0.30	(0.06–0.88)	1	2.6	0.39	(0.01–2.18)	2	7.4	0.27	(0.03–0.97)
Non-melanoma skin cancer (191)	17	20.1	0.85	(0.49–1.35)	5	3.4	1.49	(0.48–3.48)	12	16.7	0.72	(0.37–1.25)
Brain nervous system (193)	8	6.9	1.16	(0.50–2.28)	6	3.6	1.68	(0.62–3.66)	2	3.4	0.60	(0.07–2.15)
Endocrine glands (195)	2	2.6	0.78	(0.09–2.80)	1	1.2	0.80	(0.02–4.48)	1	1.3	0.75	(0.02–4.18)
Connective tissue muscle (197)	3	1.9	1.56	(0.32–4.55)	1	0.8	1.26	(0.03–7.04)	2	1.1	1.76	(0.21–6.36)
All haematopoietic cancer (200–209)	18	24.6	0.73	(0.43–1.16)	3	9.7	0.31	(0.06–0.90)	15	14.9	1.01	(0.56–1.66)
Malignant non-Hodgkin lymphoma (200)	6	8.8	0.68	(0.25–1.48)	1	2.8	0.35	(0.01–1.97)	5	6.0	0.83	(0.27–1.94)
Hodgkin’s disease (201)	2	1.4	1.39	(0.17–5.00)	1	1.0	0.97	(0.02–5.42)	1	0.4	2.41	(0.06–13.40)
Multiple myeloma, plasmocytoma (203)	5	4.2	1.18	(0.38–2.75)	0	1.7	0.00	(0.00–2.15)	5	2.6	1.96	(0.64–4.57)
Leukemia (204–207)	3	8.0	0.38	(0.08–1.10)	1	3.4	0.29	(0.01–1.62)	2	4.6	0.43	(0.05–1.59)

^a^Observed number of cancer cases

^b^Expected number of cancer cases based on cancer incidence among all men living in Stockholm County

^c^Standardized Incidence Ratio

Table 3 presents the cancer incidence for total cancer, stomach cancer and prostate cancer among firefighters in the entire follow-up, stratified by age, employment duration and starting year of employment. We only included the cancer sites that showed significantly increased or decreased risks in the entire follow-up, although malignant melanoma of the skin was omitted since there were too few cases for stratification. A trend was found for employment duration where firefighters who had been employed 1–9 years had a significantly lower cancer incidence overall (SIR = 0.47 95% CI 0.30–0.75) compared to the Stockholm reference population. Firefighters who had been employed for 20–29 or ≥ 30 years had a SIR of 0.98 (95% CI 0.77–1.26) and 0.84 (95% CI 0.72–0.98). The trend of increasing cancer incidence overall with increasing employment duration was tested statistically significant with a *p-*value of 0.03. There was an overall low risk of total cancer for all age groups, with the lowest risk in the youngest age group and then steadily increasing risk with increasing age. Firefighters younger than 50 years had a SIR for overall cancer of 0.40 (95% CI 0.15–0.86) while firefighters 65 years or older had almost the same risk as the reference population (SIR = 0.92 95% CI 0.80–1.05). There was a statistically significant trend (*p* < 0.01) of higher risk for cancer for firefighters employed early (1902–1939) compared to firefighters employed later (1940–1959, 1960–1983) but the risk was not increased in any of the categories of starting year of employment.

**Table 3:** Cancer incidence in Stockholm firefighters 1958–2012 stratified by age, employment duration and starting year of employment

	Person-years	Total cancer				Stomach cancer				Prostate cancer			
		Obs^a^	Exp^b^	SIR^c^	95% CI	Obs	Exp	SIR	95% CI	Obs	Exp	SIR	95% CI
Age (years)													
< 50	15,253	6	15.1	0.40	(0.15–0.86)	2	0.6	3.18	(0.39–11.49)	1	0.2	4.24	(0.11–23.60)
50–64	12,144	48	83.6	0.57	(0.42–0.76)	8	3.4	2.38	(1.03–4.70)	10	19.9	0.50	(0.24–0.92)
≥ 65	9093	211	229.1	0.92	(0.80–1.05)	17	10.3	1.65	(0.96–2.65)	49	68.3	0.72	(0.53–0.95)
*P* trend				*<* *0.01*				*0.07*				*0.52*	
Employment duration (years)													
1–9	8612	18	38.0	0.47	(0.30–0.75)	0		-	-	7	11.0	0.64	(0.30–1.33)
10–19	7857	20	30.4	0.66	(0.42–1.02)	2	1.0	2.02	(0.50–8.06)	3	7.4	0.41	(0.13–1.26)
20–29	8542	64	65.1	0.98	(0.77–1.26)	7	3.4	2.03	(0.97–4.26)	17	16.1	1.06	(0.66–1.70)
≥ 30	11,479	163	194.4	0.84	(0.72–0.98)	18	8.8	2.05	(1.29–3.26)	33	54.1	0.61	(0.43–0.86)
*P* trend				*0.03*				*0.19*				*0.75*	
Starting year of employment													
1902–1939	6706	116	115.0	1.01	(0.84–1.21)	15	8.3	1.81	(1.09–3.01)	24	27.4	0.87	(0.59–1.31)
1940–1959	12,912	122	135.9	0.90	(0.75–1.07)	8	4.5	1.77	(0.88–3.55)	31	35.7	0.87	(0.61–1.23)
1960–1983	16,872	27	76.9	0.35	(0.24–0.51)	4	1.5	2.72	(1.02–7.26)	5	25.4	0.20	(0.08–0.47)
*P* trend				*<* *0.01*				*0.69*				*<* *0.01*	

Italic values indicate *P* trend; < 0.05 was considered statistically significant

^a^Observed cancer cases

^b^Expected cancer cases

^c^Standardized Incidence Ratio

Firefighters showed an increased risk of stomach cancer in all ages, for employment duration over 10 years and for all starting years of employment. There was no trend of increasing stomach cancer incidence with later starting year of employment (*p-*value = 0.69) and no trend of increasing stomach cancer incidence with increasing employment duration (*p-*value = 0.19). Younger firefighters had a tendency of higher risk of stomach cancer than older firefighters.

There was no trend of increasing prostate cancer incidence with increasing employment duration (*p-*value = 0.75). Firefighters 50–64 years of age had a significantly lower risk of prostate cancer (SIR = 0.50 95% CI 0.24–0.92) as did firefighters 65 years or older (SIR = 0.72 95% CI 0.53–0.95) compared to the reference population. Firefighters employed 1960–1983 had a lower risk of prostate cancer (SIR = 0.20 95% CI 0.08–0.47) compared to firefighters employed earlier.

## Discussion

This study showed that firefighters in Stockholm had an overall low risk of cancer but an increased risk of stomach cancer compared to the reference population. Prostate cancer and malignant melanoma of the skin showed significantly low risks. There was an increasing overall risk of cancer with increasing employment duration but there was no excess of cancer overall in any of the categories of employment duration. The extended follow-up (1987–2012) provided essentially the same conclusions as the full follow-up (1958–2012).

The overall low risk of cancer in Stockholm firefighters found in this study is in contrast with many previous studies on firefighters where a slightly increased risk have been found (Daniels et al. 2014; Glass et al. 2016; IARC 2010; Pukkala et al. 2014). The low cancer risk among firefighters found in this study could potentially be explained by a selection of healthier individuals to the occupation, the so called healthy worker bias. All men working as firefighters in Sweden must pass a mandatory physical test at start of employment as well as regularly throughout their employment, which most likely makes them in better physical condition and health than the general population in Sweden (Arbetsmiljöverket 2005). However, this is also mandatory in other countries where increased risks among firefighters have been shown. The overall low cancer risk for this cohort of firefighters could also possibly be explained by the left truncation bias. The enrollment to the cohort started in 1931 but the outcome could only be studied from 1958, and therefore all firefighters who died or emigrated between 1931 and 1958 were excluded from the cohort, leaving a slightly healthier population of firefighters at start of follow-up. A sub-analysis of only firefighters employed after 1958 showed a lower overall cancer risk (SIR = 0.34 95% CI 0.23–0.49) compared to the entire cohort (SIR = 0.81 95% CI 0.71–0.91). The interpretation of this result is a bit difficult. A lower SIR indicates that there is not a left truncation bias in the overall results; however, the later population is much younger and would be expected to have a lower SIR than the older population according to our analyses stratified on age.

Even though the overall cancer risk was low, the risk increased with increasing employment duration. Analysis showed a significant trend (*p-*value = 0.03) for increasing risk of overall cancer with increasing employment duration, indicating that carcinogenic exposure at work could be of importance for the cancer risk among firefighters. The increasing overall cancer risk with increasing age and with earlier starting year of employment could also possibly be related to carcinogenic exposure since age, starting year of employment and employment duration are highly correlated in this study. However, there was no excess of cancer overall in any of the categories of age, starting year of employment or employment duration, compared to the general population.

This study showed an increased risk of stomach cancer (SIR = 1.89 95% CI 1.25–2.75). The increased risk is in line with Tornlings results from 1994 based on the same material (Tornling et al. 1994). Looking only at the extended follow-up, there was also an increased risk of stomach cancer, although not statistically significant (SIR = 1.35 95% CI 0.54–2.78). This indicates that the increased risk of stomach cancer is not just a chance finding. Tornling et al. also found in the former follow-up that the risk of stomach cancer increased with increasing number of fires the firefighters fought, which might indicate that the occupational exposure is involved in the etiology (Tornling et al. 1994). Unfortunately, in this extended follow-up with updated employment information until 2012, it was not possible to update the information on number of fires fought. LeMasters review states that firefighters are at a “possible” risk of stomach cancer (LeMasters et al. 2006). Known risk factors for stomach cancer are helicobacter pylori infection, occupational exposure in rubber production industry, tobacco smoking and ionizing radiation (Cogliano et al. 2011). There is also some evidence indicating that asbestos, lead compounds, nitrate, pickled vegetables and salted fish are risk factors for stomach cancer (Cogliano et al. 2011). A recent review and meta-analysis by Lee et al. showed a significant association between occupational crystalline silica exposure or silica-related working conditions and stomach cancer (Lee et al. 2016). It is possible that firefighters are exposed to rubber compounds if the burning material consists of these compounds or asbestos and crystalline silica dust if the fire site holds these materials. An increased risk of lung cancer and pleura cancer/mesothelioma would be expected if firefighters were exposed to asbestos. This was not shown for lung cancer in our material (SIR = 0.79 95% CI 0.52–1.15) but for pleura cancer we observed 2 cancer cases compared to 0.8 expected cases. This might indicate an increased risk for pleura cancer although the numbers are too few to draw any conclusions. A review from Raj et al. concluded that “dusty occupations” could be a cause of stomach cancer (Raj et al. 2003). Firefighters often get in contact with dust when tearing down burning material, and during the last stage of the firefighting (overhaul).

The decreased risk of prostate cancer found in firefighters in this study is somewhat surprising since several previous studies and meta-analyses have found an increased risk of prostate cancer in firefighters and since shift work possibly could increase the risk of prostate cancer (IARC 2010). It is possible that Swedish firefighters are not disturbed as much during shift and night work compared to firefighters in other countries, with less impact on the circadian rhythm, although we do not have such information.

Firefighters in this study had no observed cases of testicular cancer, with 1.5 cases expected. Both LeMasters et al. and IARC found an increased risk of testicular cancer (IARC 2010; LeMasters et al. 2006). However, the Nordic study NOCCA found in 2014 a significantly decreased risk of 0.51 (95% CI 0.23–0.98) for testicular cancer (Pukkala et al. 2014). Also a recent study on 30,000 US firefighters followed from 1950 to 2009 found a decreased testicular cancer incidence (Daniels et al. 2014). These two studies make it less likely that our finding is due to chance. The risk factors for testicular cancer are mainly unknown, and so is the reason for a reduced risk among firefighters.

This study has several strengths and weaknesses. This is a cohort study with a long follow-up time in the cancer registry of up to 54 years. This long follow-up allows for cancer incidence to be studied. Cancer incidence was collected from the Swedish Cancer Registry with a high coverage of 96%. Unfortunately, there were no individual data for lifestyle factors like tobacco smoking available and therefore analyses could not be adjusted for potential confounders. Firefighters’ employment duration was used as a proxy for their exposure which is a weakness and no information on the use of respiratory protection and other protective equipment was available. It should also be considered that this is an occupational cohort and that the reference population is the Stockholm population. This can increase the risk of bias due to a selection of healthy individuals to the occupation.

The generalizability of this study is thought to be high in Sweden, but could be lower internationally due to differences in the firefighting occupation between countries, such as type of work activities, type of materials that may burn, methods of fire extinguishing and use of protective equipment. The exposure to carcinogens may also differ between work in urban and rural locations and between different periods of time (Fritschi and Glass 2014). Smoking habits may differ between countries but a recent study on lung cancer among firefighters in Europe, Canada, New Zeeland and China showed that smoking was similarly common in firefighters as in non-firefighters (Bigert et al. 2016). The diagnostic criteria between countries are not likely to differ; however, the reporting of cancer cases could differ as Sweden has a mandatory cancer register with a very high coverage of 96% (Barlow et al. 2009).

## Conclusion

This study showed that Stockholm firefighters had an overall low risk of cancer. However, firefighters had an increased risk of stomach cancer compared to the reference population. There was an increasing overall risk of cancer with increasing employment duration, which potentially could be related to the carcinogenic exposures at work, although there was still no excess of cancer overall in any of the categories of employment duration.
